# Interactive Field Effect of Atomic Bonding Forces on the Equivalent Elastic Modulus Estimation of Micro-Level Single-Crystal Copper by Utilizing Atomistic-Continuum Finite Element Simulation

**DOI:** 10.3390/molecules25215107

**Published:** 2020-11-03

**Authors:** Chang-Chun Lee, Jing-Yan He

**Affiliations:** 1 Department of Power Mechanical Engineering, National Tsing Hua University, No. 101, Section 2, Kuang-Fu Road, Hsinchu 30013, Taiwan; 2 Department of Mechanical Engineering, National Chung Hsing University, No. 145 Xingda Rd., South Dist., Taichung 40227, Taiwan; hjy2018@foxmail.com

**Keywords:** atomistic-continuum method, morse potential, atomic bond, finite element analysis, elastic modulus

## Abstract

This study uses the finite element analysis (FEA)-based atomistic-continuum method (ACM) combined with the Morse potential of metals to determine the effects of the elastic modulus (*E*) of a given example on atomic-level single-crystal copper (Cu). This work aims to overcome the estimated drawback of a molecular dynamic calculation applied to the mechanical response of macro in-plane-sized and atomic-level-thick metal-based surface coatings. The interactive energy of two Cu atoms within a face-centered metal lattice was described by a mechanical response of spring stiffness. Compared with the theoretical value, the parameters of the Morse potential dominated the predicted accuracy through the FEA-based ACM. Moreover, the analytic results indicated that the effective *E* of a single-crystal Cu was significantly sensitive to the given range of the interactive force field among atoms. The reliable elastic moduli of 86.8, 152.6, and 205.2 GPa along the Cu(100), Cu(110), and Cu(111) orientations of the Cu metal were separately acquired using the presented FEA-based ACM methodology.

## 1. Introduction

With the rapid development and widespread utilization of flexible optoelectronic devices, macro in-plane metal-based surface coatings with an atomic-level thickness and nanowires composed of single-crystal copper (Cu) are being utilized as electrodes because of their outstanding electronic and mechanical responses [1,2]. The mechanical properties of the Cu metal vary significantly from the macroscale to the nanoscale. In addition, the orientation of crystal lattices is influenced by anisotropy behavior. Consequently, the foregoing mechanical properties of thin films scaled down to the nanoscale or atomic level are difficult to acquire through experiments. In previous work, the elastic moduli of single-crystal Cu at the lattice orientations of (100), (110), and (111) were reported as 66.7, 130.3, and 191.1 GPa, respectively [3]. The abovementioned values are estimated by elasticity solid mechanics with the integration of measured stiffness and compliance constants. Compared with the analytical solution, smaller values were measured by microcantilever testing for single-crystal Cu along the abovementioned lattice orientations (i.e., 43–59, 123–145, and 148–181 GPa, respectively) [4]. Wang et al. [5] adopted nanoindentation to measure the directional modulus of thin Cu coatings. However, compared with the testing data of other research groups, an obvious deviation was noted on the (100) orientation because of an elastic modulus (*E*) in the range of 115–142 GPa. Dub et al. [6] utilized a similar approach and obtained significantly different testing results because magnitudes of 52 and 170 GPa were obtained for the Cu(100) and Cu(111) directions, respectively. Thus, numerous tests have been conducted to assess the anisotropic mechanical properties at the nanoscale or atomic level. However, the experimental procedures for atomistic-scale coating, which can induce geometry/size effects, still present serious deviations, even if testing preparations were made. Some mechanical properties lead to the predicted difficulty of device reliability. Thus, simulation has been adopted to obtain the nanoscale mechanical properties of ultrathin metal coatings to eliminate geometric complexity and measured uncertainty. Molecular dynamics (MD) is one of the popular numerical methods for predicting the properties of atomic-scale metals. Ahadi et al. [7] employed MD by applying a finite tensile strain to investigate the size-dependent Poisson’s ratio of crystal Cu nanobeams having crystallographic orientations. Moreover, the yield point and directionality of Cu nanotubes under a three-axial tension can be estimated through MD [8]. Similar numerical procedures were used to extract the temperature-dependent plastic deformation of Cu nanowires when the related stress–strain curve was completely acquired [9]. The finite element analysis (FEA)-based atomic-continuum method (ACM) is a promising alternative to MD simulation for studying materials emulating mechanical properties, such as carbon nanotubes (CNTs), as the latter require intensive calculations. Li et al. [10] were the first to propose that the interaction between a pair of carbon atoms linked by a chemical bond could be equivalent to a beam with two nodes. Using ACM theory, Tserpes et al. [11] used FEA to calculate the Young’s modulus of a single-wall CNT that replaced the covalent bonds between carbon atoms with a beam element having six degrees of freedom. Previous studies on ACM simulation used spring elements to explore the shear modulus and vibration [12,13,14,15]. The abovementioned approaches are widely adopted in the investigation of different materials, especially material characteristics highly dependent on the lattice or microstructure. Cu, one of the widely utilized materials in semiconductor processing, also has many characteristics that need to be explored by the utilization of MD and FEA-based ACM approaches. The plasticity and void growth behavior of copper are enabled by atomistic modeling and statistical analysis [16,17]. Moreover, some publications are focused on the estimation of the fracture properties of single crystal Cu by similar approaches. Lee et al. [18] used the interatomic potential function of the embedded-atom method to model face-centered cubic structures and to analyze the mode-I fracture behavior under various magnitudes of T-stress. The fractal theory is adopted to interpolate the surface roughness and morphology influence between the Cu rigid plane and elasto-plastic rough substrate [19]. Furthermore, the mixed-mode fracture characteristics of single crystal Cu are revealed to induce the transition of fracture patterns as the incensement of fracture mode mixity [20]. Compared with general atomistically calculated approaches such as MD, the FEA-based ACM method requires fewer computing resources and satisfies the requirements of effective material properties applied to large simulated models. Consequently, the mechanical properties estimated using FEA-based ACM may be related to the characteristics of thin films constructed in a global device model for reliability predictions and are beneficial for design improvements, such as the flexibility of device applications. As previously mentioned, the modeling scope of the MD simulation, which considers the bonding interactions among the considered atoms, is quite small (less than 10,000) because of hardware limitations, such as central processing unit and memory. Thus, MD calculation is unsuitable for emulating the effects of atomic-level coating on an entire structure within a large-scale estimated model. To resolve this problem, the extraction of the *E* of atomic-level single-crystal Cu through FEA-based ACM is proposed. Given that the considered range of the interactive force field among Cu atoms is extended to a lattice length of 3.61 Å, several kinds of spring stiffnesses, adopted in the ACM model, are necessary to describe the effects of the different levels of bonding strength on the atomic-level mechanical characteristics. Thus, the bonding effect among atoms at neighboring levels on the estimated effective *E* of atomic-scale Cu metal was discussed in detail. In addition, five sets of Morse potential parameters, used to describe the bonding behavior of Cu atoms, were examined for their predicted accuracies on the effective modulus.

## 2. Potential Function of Cu Metal Atoms

### 2.1. Morse Potential

The Morse potential is typically utilized because of its general suitability for metallic bonds and simple formula structure for various fields, such as nanoindentation and machine tool cutting optimization [21,22,23,24,25]. In accordance with Girifalco and Weizer’s demonstration for calculating second-order elastic constants [26], the Morse potential is useful to the atomic properties of metals. The interaction energy *V_ij_* of a pair of atoms *i* and *j* is expressed as:

(1) $$V_{ij}=D\left\{\exp\left[-2\alpha \left(r_{ij}-r_{0}\right)\right]-2\exp\left[-\alpha \left(r_{ij}-r_{0}\right)\right]\right\}$$

where *r_ij_* is the distance between the atoms *i* and *j*. The symbols *D* and *α* represent the depth of the interatomic potential well and the force constant, respectively. The index *r*_0_ is the equilibrium distance between the atoms *i* and *j*. The interatomic force *F* of pairwise atoms can be obtained by derivation with respect to the distance *r_ij_*. Once again, the second derivative of Equation (1) was implemented to gain the stiffness *k* of two isolated atoms of *i* and *j*. In this way, the effective spring constant adopted in ACM to describe the behavior of atoms was found. The formulas for the interatomic force *F* and the stiffness *k* are shown in Equations (2) and (3), respectively:

(2) $$-F=\frac{\partial V_{ij}}{\partial r_{ij}}=-2\alpha D\left\{\exp\left[-2\alpha \left(r_{ij}-r_{0}\right)\right]-\exp\left[-\alpha \left(r_{ij}-r_{0}\right)\right]\right\}$$

(3) $$k=\frac{\partial^{2}V_{ij}}{\partial r_{ij}^{2}}=2\alpha^{2}D\left\{2\exp\left[-2\alpha \left(r_{ij}-r_{0}\right)\right]-\exp\left[-\alpha \left(r_{ij}-r_{0}\right)\right]\right\}$$

### 2.2. Comparisons of Selected Morse Parameters

**Figure 1:**
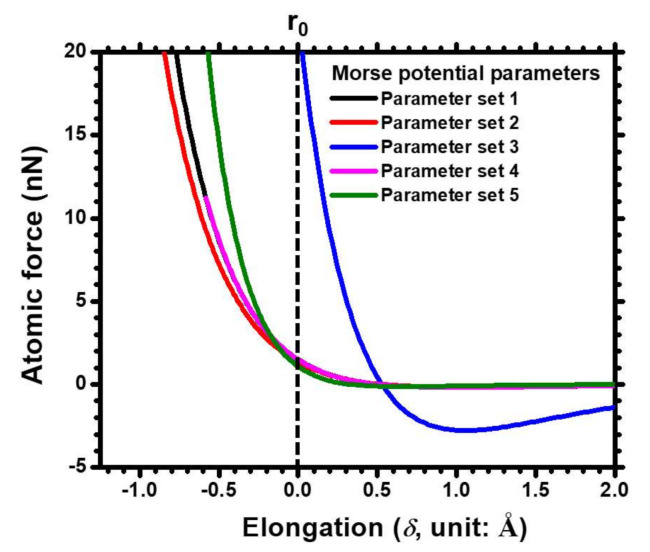
A force-elongation (*δ*) curve of the Cu-Cu atomic bond calculated by the Morse parameters of the present five sets (1 Å = 10^−10^ m).

To achieve a good estimated result of the Cu’s *E* by using FEA-based ACM combined with the Morse potential functions, suitable and accurate Morse parameters must be determined. Consequently, five parameter sets of Morse parameters for Cu metal were obtained from different research groups to determine their similarity with the theoretical value and the measured data. Set 1 was provided by Girifalco et al. [26] and can extract the elastic constants for face-centered and body-centered metals, including Au, Ag, and Cu. The constants were calculated by using experimental values, such as the vaporization energy, lattice constant, and compression. Set 2 was obtained from Cotterill et al. [27]. The atom arrangement at the dislocated edge was depicted by central-force approximation with a reflected truncation. Various energy functions, including the Morse potential, were used to calculate the stacking-fault energy, which was highly dependent on the potential form and truncated dimension. In set 3, the relevant constants were based on the second-order and third-order elastic constants of cubic metals such as Cu, Ag, and Al [28]. The interaction of atoms was described based on the Morse parameters, which were determined based on the physical characteristics of the bulk modulus and cohesive energy. Another set of potential constants was obtained from a nanoscale machining study of tool wear and fraction [29]. In set 4, MD combined with the Morse potential was used to calculate the friction of dissimilar materials at the tool–work interface. Similarly, the Morse parameters of set 5 were subjected to an MD simulation to explore the phonon dispersion curves and the dependence of the Debye temperature for Cu lattices [30]. The calculated results agreed well with the experimental data. Table 1 lists the Morse parameter sets from these five resources. Moreover, the relations between the applied force and elongation (*δ*, with respect to r_0_) of a pair of Cu atoms on the basis of the corresponding Morse parameters are shown in Figure 1.

## 3. Atomistic-Continuum Method in FEA Modeling

**Figure 2:**
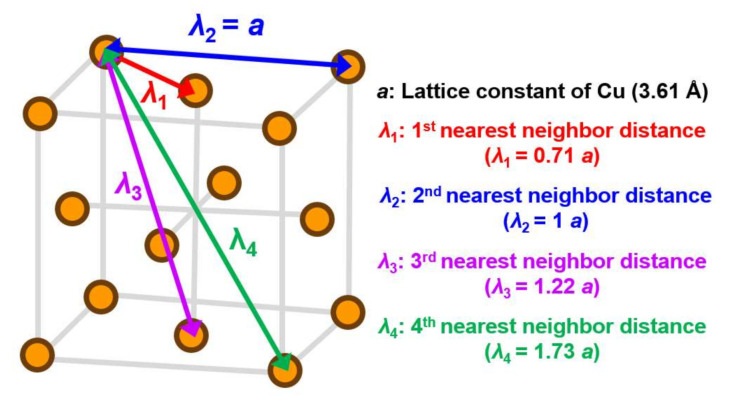
A diagram of the bonding distance from the 1st to the 4th nearest-neighbor distances between atoms (*λ*_1,_ *λ*_2,_ *λ*_3,_ and *λ*_4_) within a face-centered lattice cell unit.

**Figure 3:**
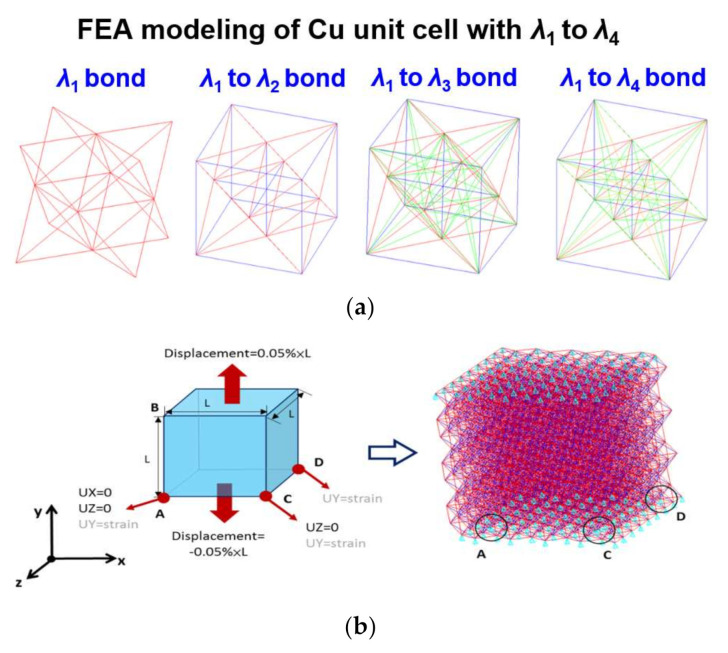
The atomic-level FEA modeling of single crystal Cu: (**a**) Modeled node (atom) and interactive bonding between atoms, considering first to fourth nearest neighbor atoms’ bonding; (**b**) Boundary conditions of single crystal Cu with a cube having a length of 30 Cu lattice constants (3.61 Å) when a 0.1% tensile uniaxial strain is loaded.

As mentioned in the previous section, a FEA-based ACM was used in this study. The simulation work is performed by the commercial FEA software ANSYS 18.0. The fundamental concept of ACM is that the atoms and bonding force are replaced by nodes and springs with varying stiffnesses. For single-crystal Cu, a 3D unit cell with a face-centered arrangement of atoms was constructed in order to form a finite element model with the required size scope. A schematic diagram of single crystal Cu with different levels of the nearest neighbor atoms is illustrated in Figure 2. To complete the atomic-level modeling of single-crystal Cu, the procedure flow is described below. First, the node was considered as atoms and, in order to build the cubic single crystal structure of Cu, its lattice constant was 3.61 Å, this distance being defined as the symbol *a*. For single crystal Cu, the four nearest neighbor atoms were totally contained in its cubic structure. The distance between and the first nearest (*λ***_1_**) neighbor atom was 2.56 Å (0.71 *a*), and that between any Cu atom and the second nearest (*λ*_2_) neighbor atoms was 3.61 Å (*a*), which was equal to the lattice constant of Cu itself. The distances of the third nearest (*λ*_3_) and fourth nearest (*λ*_4_) neighbor atoms were 4.43 (1.22 *a*) and 6.26 Å (1.73 *a*), respectively. As the interaction of Cu atoms has a form similar to a force field, the effective range is considered from the first to the fourth nearest neighboring atom and has a cut-off radius of 6.26 Å. In the present FEA-based ACM procedure, the spring elements have an equivalent spring constant *k*, which is calculated and utilized to simulate the relationship between loads versus the displacement of atomic bonding between Cu atoms. The corresponding effective spring constants of four different neighboring levels of Cu atoms, calculated with the Morse parameters of the five sets, were calculated using Equation (3), the detailed calculation flow being described as follows. First, we adopted the constant parameters *D*, α, and *r*_0_ into Equation (3). Then, we utilized the considered *r_ij_* to calculate the corresponding effective *k*. For example, the *r_ij_* was considered as *λ***_1_** for the *k*_1_ calculation. In a similar way, the *k*_2_, *k*_3_, and *k*_4_ could be separately estimated. It is worth mentioning that the foregoing *k* values are in eV/ (Å^−1^)^2^. With the unit conversion from eV to nN·Å (1 eV = 1.602 × 10^−19^ J = 1.602 × 10^−19^ N·m = 1.602 nN·Å), the final estimated *k* values in the nN/Å unit can be obtained and are summarized in Table 2. Following the abovementioned procedure, the atomic-level FEA modeling of single crystal Cu, considering the first to fourth nearest neighbor atoms’ bonding and its boundary conditions for the tensile test, is illustrated in Figure 3. Figure 3a represents the modeled atoms (nodes) and interactive bonding between the Cu atoms of the FEA model. In addition, the boundary condition setting is shown in Figure 3b, with a 0.5% tensile strain being loaded at the top and bottom surface of the Cu crystal. That is to say, a 0.1% tensile strain is applied to extract the effective *E* from a Cu crystal with different size domains. In this study, the lattice planes of (100), (110), and (111) were chosen as the loaded orientations during the modeling process. After the tensile strain was induced, the reaction forces were captured in order to calculate *E* in accordance with the generalized Hooke’s law.

## 4. Results and Discussion

### 4.1. Estimated Accuracies of the Morse Parameters

**Figure 4:**
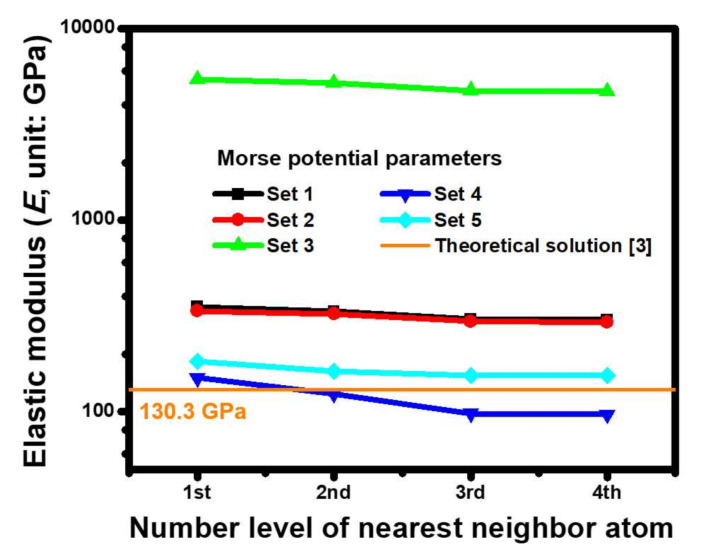
A comparison of *E* for a Cu(110) crystal cube with a length of 30 *a* (*a* = 3.61 Å), estimated by means of the Morse parameters of five sets presented in this study, as various cut-off levels of the pairwise interaction are taken into account.

To determine the Morse parameters that can be combined with the present ACM simulation and acquire good estimated results as compared with the theoretical solutions, the estimated results obtained using the parameters provided from the five sets were compared with each other. Given the selected example of the predicted results for the orientation of Cu(110) (Figure 4), the range impacts of the force field truncated among the Cu atoms are included by increasing the cut-off levels of the nearest neighboring atom under a fixed cubic volume with an edge length of 30 *a* (*a* = 3.61 Å). In other words, considering the different spring constants in the ACM model, the interactive forces among the atoms of a Cu lattice would further approach the bonding conditions of the actual Cu crystal structures. The analytic results revealed that the estimated result of 154.1 GPa, obtained by introducing the Morse parameter of set 5 into the ACM-based FEA, agreed well with the *E* of 130.3 GPa. The *E* was reduced from 182.7 GPa to 154.1 GPa when the effective spring constants within the ACM model were accumulatively considered from the first to fourth nearest neighboring atom. In addition, the ACM results of the third and fourth nearest neighboring atoms were nearly identical, implying that the cut-off was sufficiently large to calculate the effect of the atomistic force field at that moment. Consequently, the parameters of set 5 were the best among all the sets because the predicted deviations in other sets were quite evident. Based on the analytical results and discussed consequences, the subsequent results of the ACM analysis were obtained by utilizing the Morse parameters of set 5.

### 4.2. Oriented Dependence of Atomic-Scale Elastic Modulus (E) Estimated based on ACM Simulations

**Figure 5:**
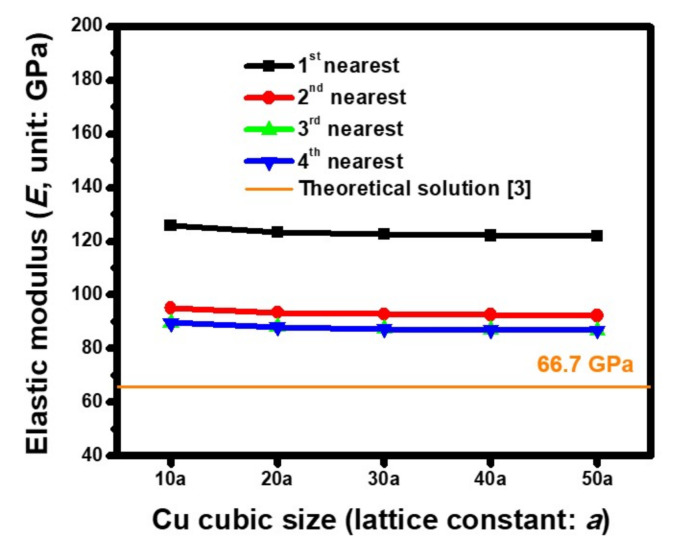
Size-dependent *E* of the Cu(100) crystal cube estimated by using the Morse parameters of set 5 when several kinds of cut-offs for interatomic pairwise interaction are considered (*a* = 3.61 Å).

**Figure 6:**
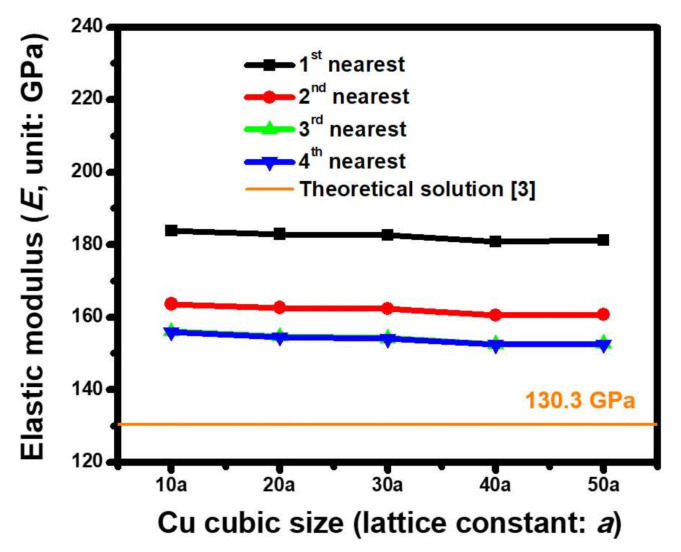
Size dependence of *E* for the Cu(110) crystal estimated by utilizing ACM-based FEA when the Morse parameters of set 5 are considered (*a* = 3.61 Å).

**Figure 7:**
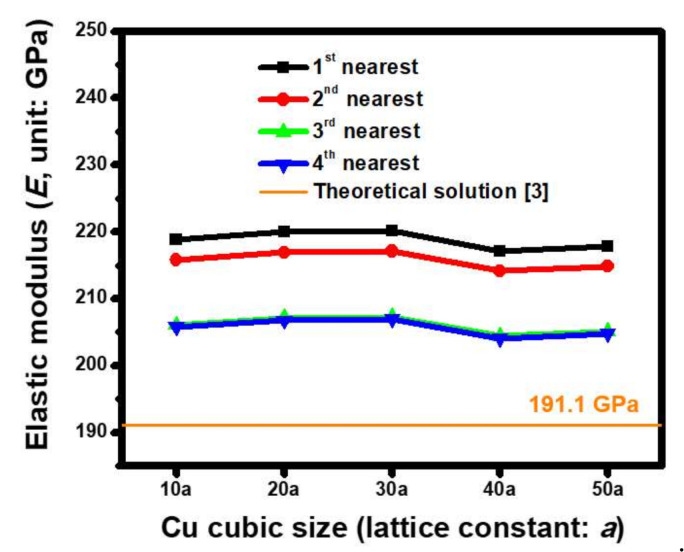
Size dependence of the estimated *E* acquired by ACM-based FEA combined with the Morse parameters of set 5 when the (111) orientation of the Cu crystal cube is introduced (*a* = 3.61 Å).

Three lattice orientations in a single-crystal Cu cube, including Cu(100), Cu(110), and Cu(111), were separately built, based on the ACM analysis, in order to extract the corresponding *E*. As shown by the estimated results of Cu(100) in Figure 5, the size dependence of the Cu crystal, under various interactive extents between atoms, on the *E* was analyzed. The Cu dimensions changed from 10 *a* to 50 *a* (*a* = 3.61 Å), which did not significantly influence the mechanical characteristics because the estimated *E* was only reduced by 3.38% from 89.71 GPa to 86.77 GPa. This phenomenon was attributed to the examined size range from 3.61 nm to 18.05 nm, which was regarded as being small at the nanoscale level. Consequently, a micro- to macrolevel size effect was excluded in this analysis. Compared to this, the force field effect was significant. When taking the example of a Cu cube with a 50 *a* (*a* = 3.61 Å) edge, the effect of the *E* was only equal to 121.81 GPa if the first nearest bonding force of a pair of atoms was considered. A magnitude of 92.37 GPa was predicted when the second nearest bonding force was included. This behavior was attributed to a fresh force equilibrium within the ACM model of the generated Cu lattice. When the balanced conditions were further enhanced by considering the other two high-order bonding forces, i.e., the third and fourth nearest interactions of atoms, the foregoing effective *E* finally converged to 86.8 GPa. Similar simulated procedures were adopted, and the estimated ACM results at the orientations of Cu(110) and Cu(111) are shown in Figure 6 and Figure 7, respectively. As shown by the converged extent of the calculated modulus in these figures, at least the first three nearest bonding forces should be considered in order to meet the requirements of the range for the interactive force field within a Cu lattice. Table 3 summarizes the estimated results obtained by ACM analysis proposed in this study and the data collected from previous work. The oriented dependence of the nanoscale effective elastic moduli extracted by ACM simulation was slightly greater than the theoretical values. The reason for such a difference was the size effect. Moreover, the measured data of the Cu crystals demonstrated that the estimated results were reasonably acceptable because the predicted magnitude was located within the distributed scope of the experimental data.

## 5. Conclusions

For a nanoscale surface coating with a large dimensional area, the corresponding reliability performance under external loadings is difficult to estimate because both the mechanical properties and numerical modeling of the atomic-scale coating must be considered. In this study, the FEA-based ACM was used to resolve this problem through a given example of atomic-level single-crystal Cu. With the assistance of the Morse potential, the interactive behavior among Cu atoms was successfully described by several kinds of spring stiffnesses within the ACM model. In other words, the *E* of the nanoscale single-crystal Cu cube was estimated using FEA by turning the interaction of pairwise atoms into an effective spring constant between two nodes. The analytic results from the ACM simulation indicated that, within an atomic scope, the elastic moduli along the lattice orientations of Cu(100), Cu(110), and Cu(111) were similar to the measured range of the experimental data and the analytical solution. Moreover, the selection of the Morse potential parameter was highly correlated with the estimated mechanical properties when compared with the simulated results when using several sets of parameters. Furthermore, the results revealed that the interactive range of Cu atoms in the ACM model should take the bonding for the first three nearest neighboring atoms into consideration in order to acquire a more stable *E* magnitude.

## Figures and Tables

**Table 1:** A list of five sets of Morse parameters considered for ACM-based FEA.

Parameter Set No.	*D* (eV)	*α* (Å^−1^)	*r*_0_ (Å)	Ref.
1	0.343	1.356	2.866	[26]
2	0.324	1.294	2.913	[27]
3	5.259	1.312	2.899	[28]
4	0.343	1.356	2.626	[29]
5	0.162	2.093	2.616	[30]

**Table 2:** Effective spring constants of the 1st to 4th nearest neighbor atoms utilized in ACM-based FEA.

Parameter Sets No.	k_1_ (nN/Å)	k_2_ (nN/Å)	k_3_ (nN/Å)	k_4_ (nN/Å)
1	6.286	−0.202	−0.185	−0.020
2	5.998	−0.135	−0.176	−0.022
3	97.090	−2.480	−2.853	−0.343
4	2.676	−0.253	−0.145	−0.014
5	3.262	−0.211	−0.049	−0.001

**Table 3:** A comparison of crystal-oriented elastic moduli for atomic-scaled Cu metal extracted from theoretical values, experimental data, and the simulated results of ACM-FEA presented in this study. (Unit: GPa).

Cu(100)	Cu(110)	Cu(111)	Ref.
66.7	130.3	191.1	Theoretical solution [3]
51~55	121~134	161~181	[4]
115~142	147~167	140~169	[5]
52	None	170	[6]
86.8	152.6	205.2	This study
